# Supplementary figures and images for: Phenotypic delay in the evolution of bacterial antibiotic resistance: Mechanistic models and their implications
Source: PLoS Comput Biol. 2020 May 29;16(5):e1007930. doi: 10.1371/journal.pcbi.1007930 (PMC7307788; doi:10.1371/journal.pcbi.1007930)

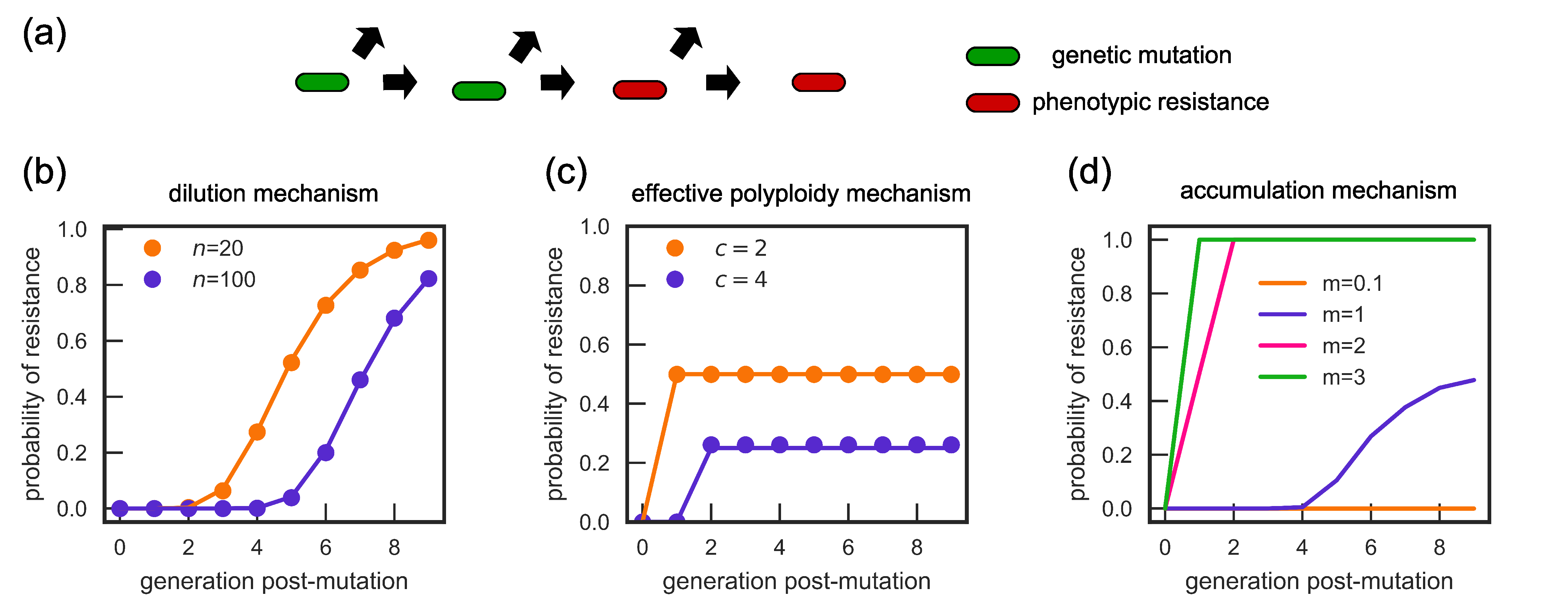

Supplement: S1 Fig — (a) We follow a single bacterium which has just mutated and has the resistant allele in one of its chromosomes. When it divides, we choose one of the two daughter cells at random. After a few generations, this cell can become phenotypically resistant. (b) The probability of the cell being resistant as a function of the number of generations from the genetic mutation for the dilution mechanism (dots: simulation, lines: theory Eq (S1)). (c) Same as (b) for the effective polyploidy mechanism. (d) Same as (b) for the accumulation mechanism (only simulations). (TIF) [file pcbi.1007930.s003.tif]

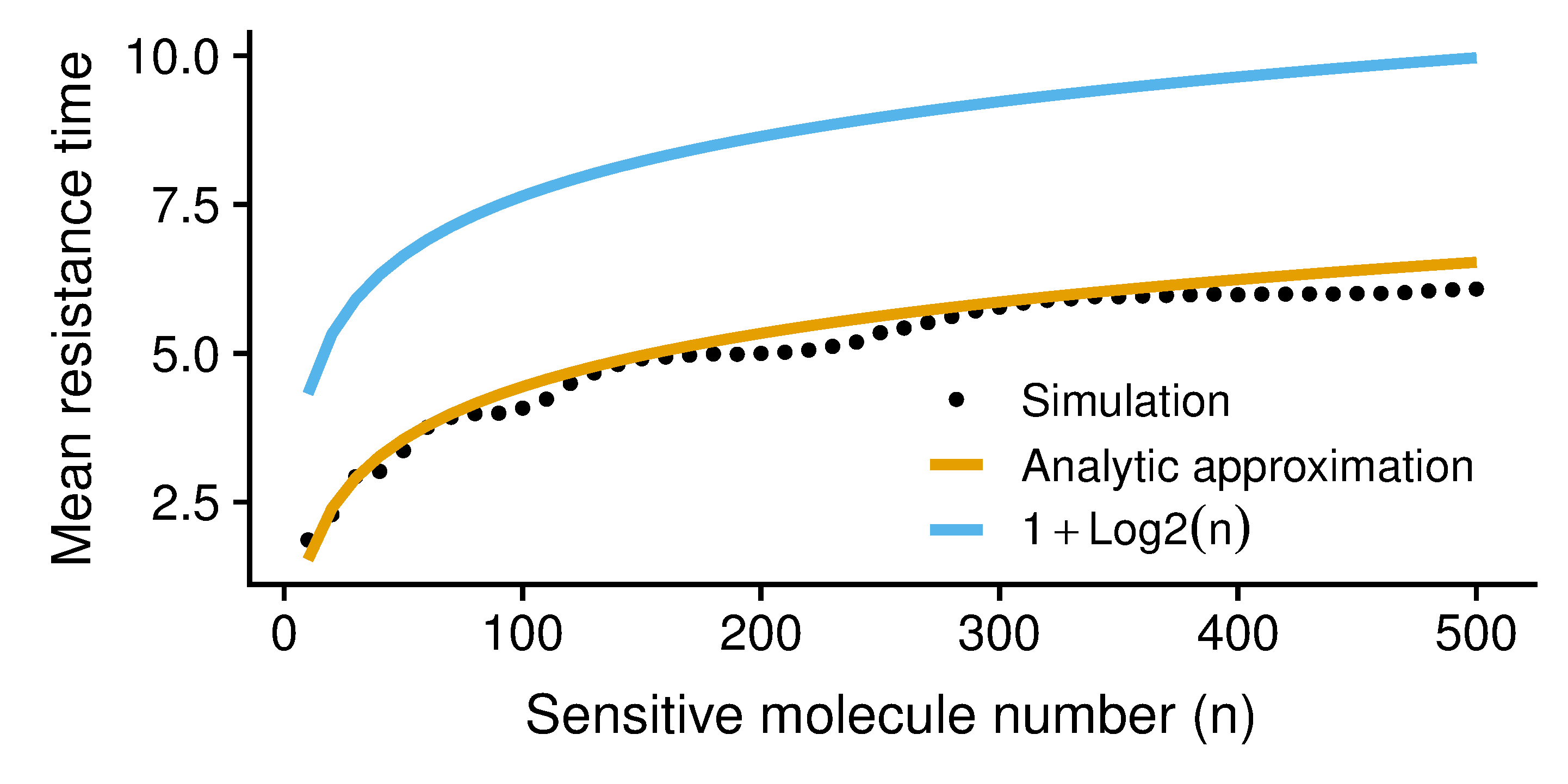

Supplement: S2 Fig — We start with x = 100 cells that just mutated, and repeat the simulation 500 times for each data point. “Analytic approximation” refers to Eq (S6). (TIF) [file pcbi.1007930.s004.tif]

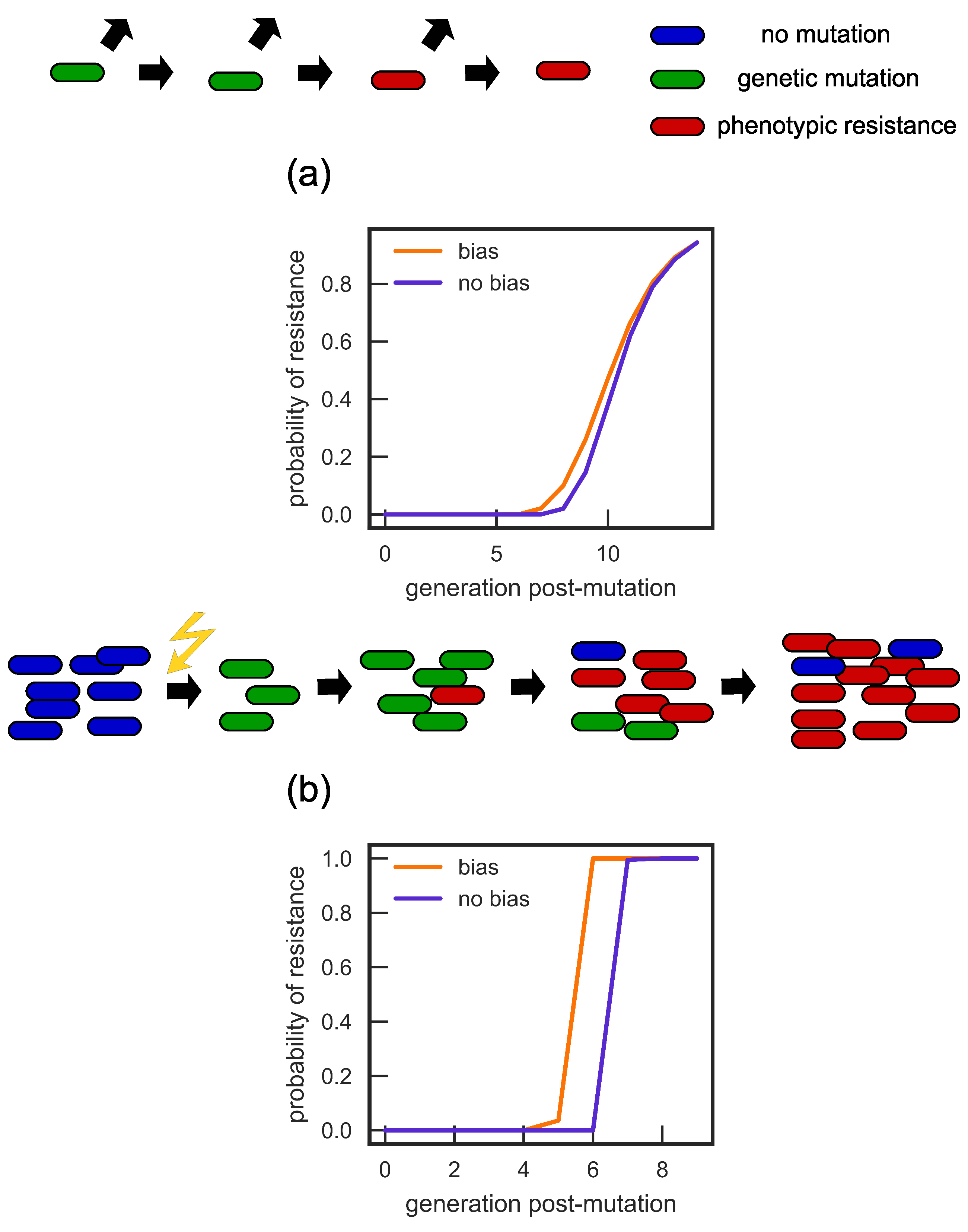

Supplement: S3 Fig — Blue curve represents an unbiased case (p = 0.5), orange curves is the biased case (p = 0.62). In all cases, n = 1000. (TIF) [file pcbi.1007930.s005.tif]

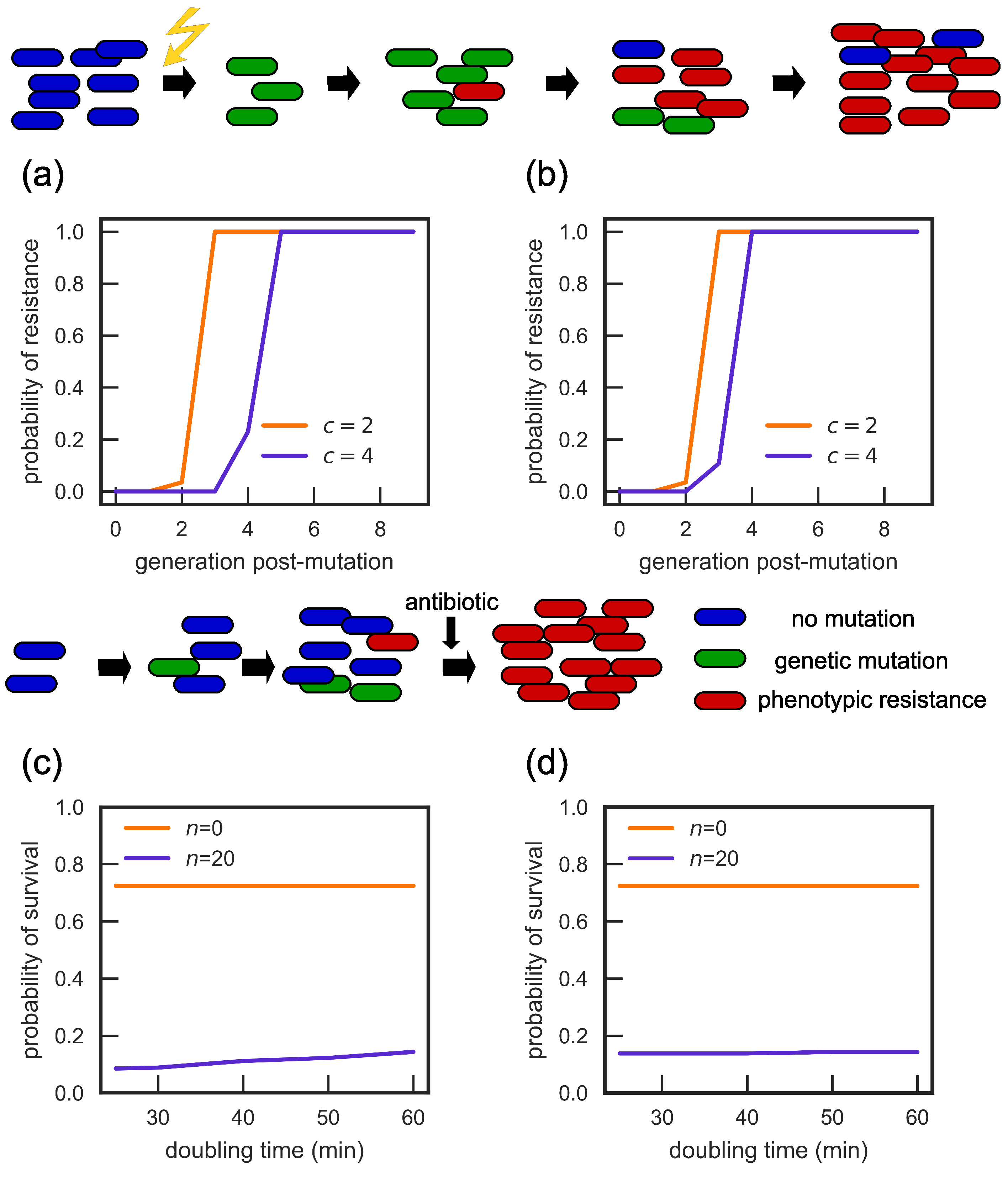

Supplement: S4 Fig — (a) Probability of resistance as a function of time (generations) for different doubling times (determined by ploidy c) when the number of target molecules n depends on td. (b) Same as (a) but for the model in which n does not depend on td. (c) Probability of survival for a simulated infection (see section 2.3 and Fig 3 in the main text) for a combined model when the number of target molecules depends on the growth rate. (d) Same as (c) but for the model in which n does not depend on td. (TIF) [file pcbi.1007930.s006.tif]

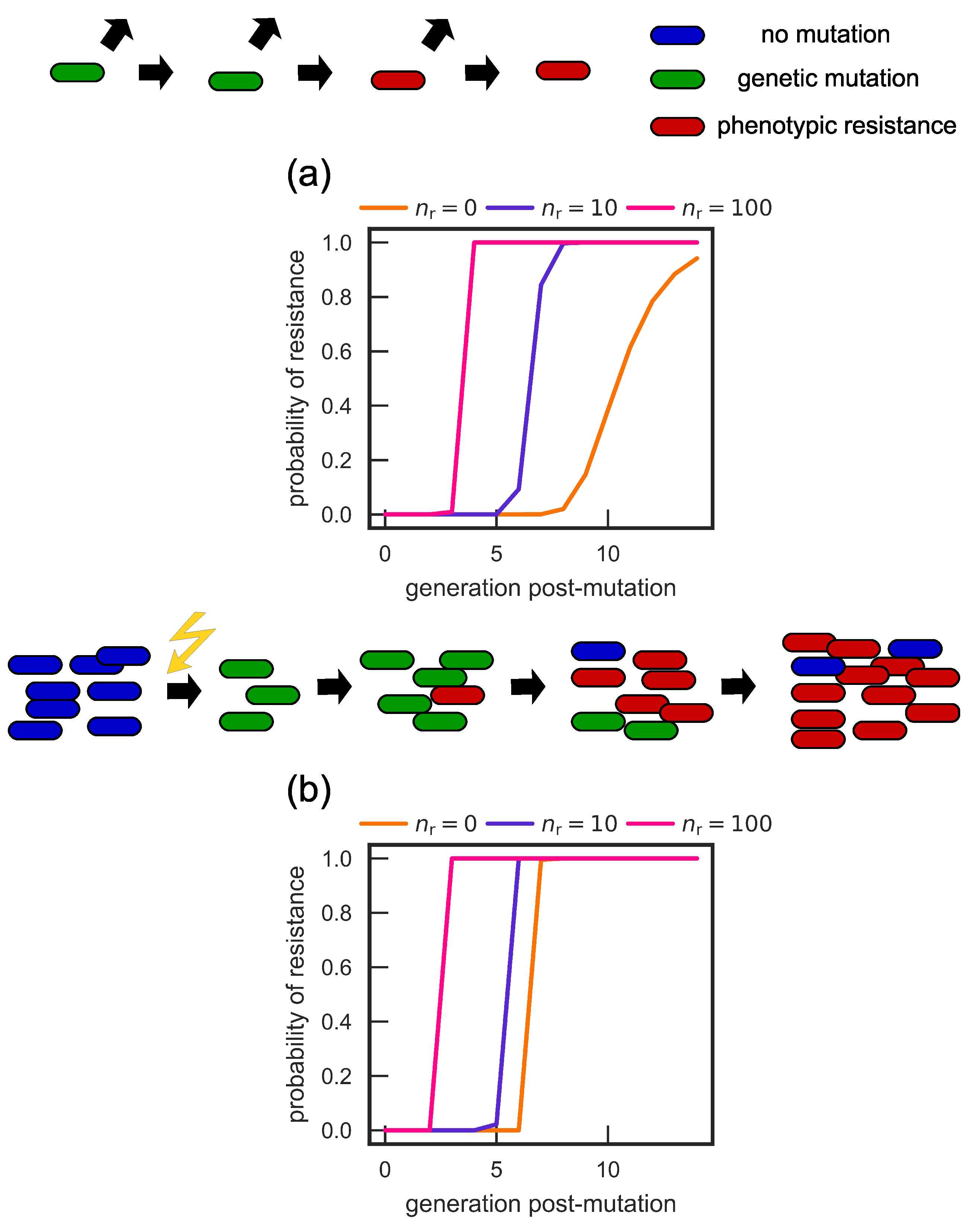

Supplement: S5 Fig — (a) Single-cell and (b) population level simulated experiments as a function of nr, the number of sensitive molecules allowed for resistance to emerge. In all cases, the total number of molecules n = 1000. (TIF) [file pcbi.1007930.s007.tif]

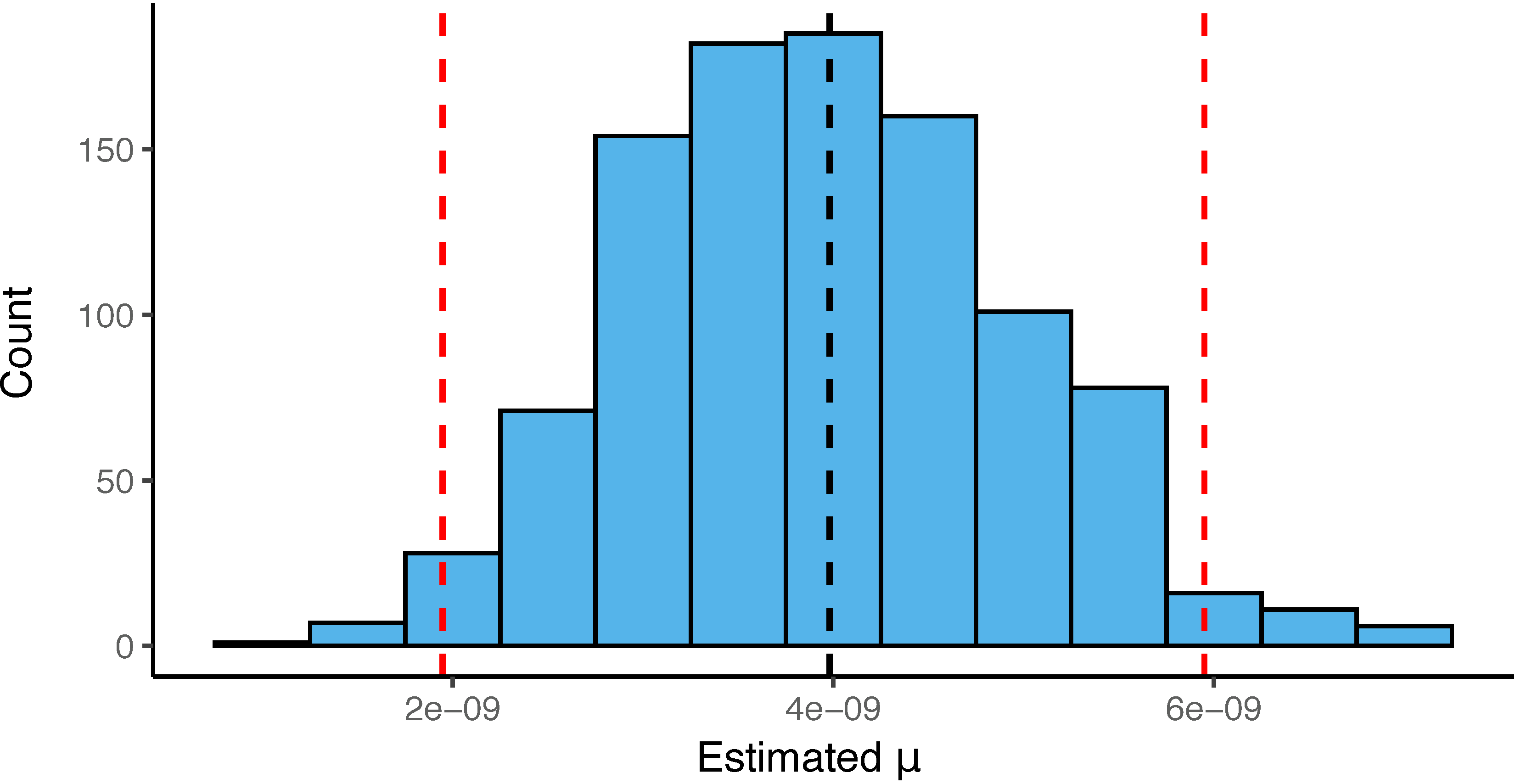

Supplement: S6 Fig — The mutation probability can be underestimated by a factor of 2 (95% of simulations yielded estimates between red vertical lines), whereas Ref. [46] reports a factor of 9.5 difference between μ obtained from DNA sequencing and fluctuation tests. The Lee et al. result [46] cannot be thus explained by the no-delay model. (TIF) [file pcbi.1007930.s008.tif]

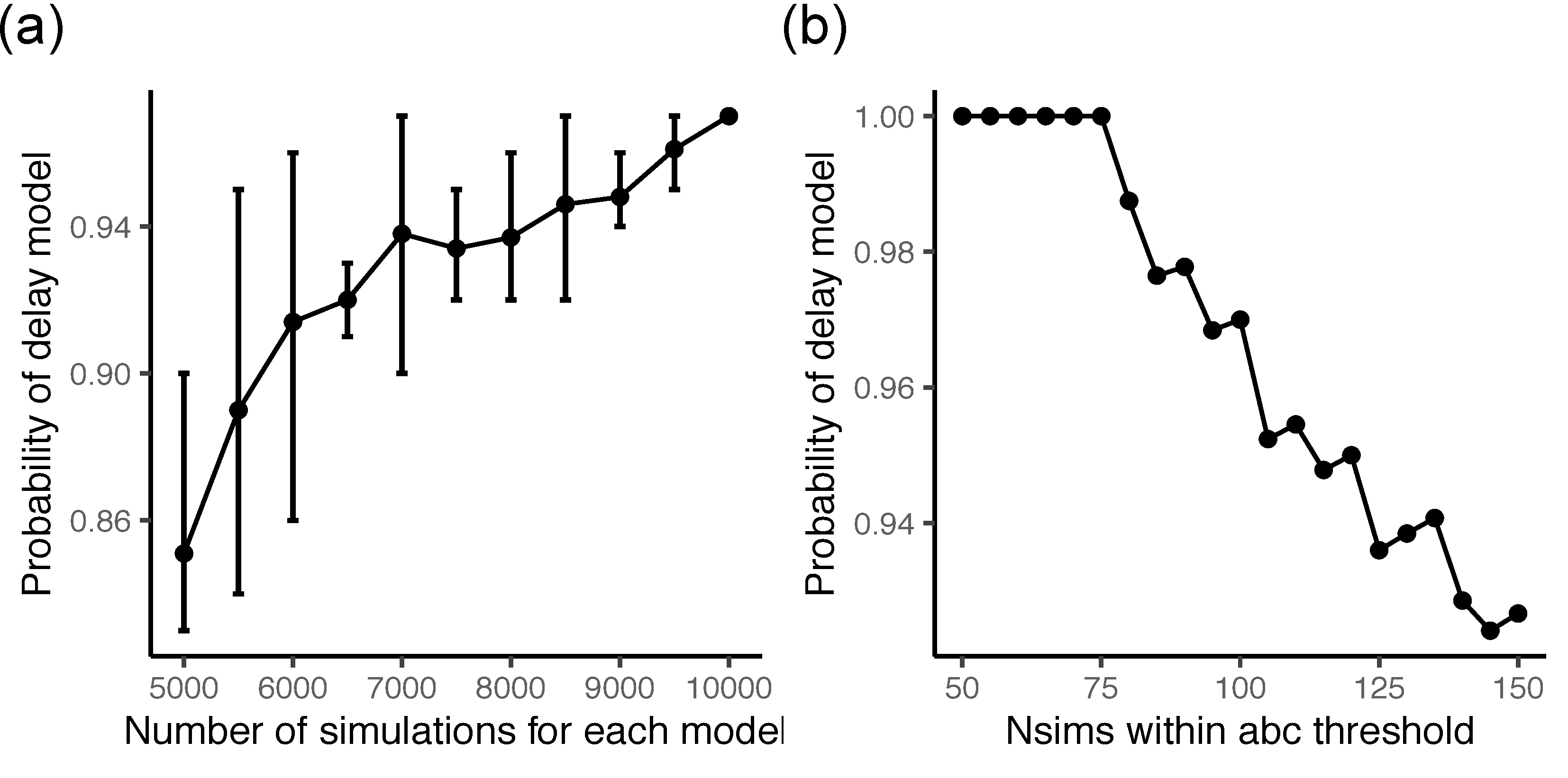

Supplement: S7 Fig — (a) The probability of the Boe et al. data [47] coming from the delay model as a function of the number of simulation runs. The runs were randomly sampled from the original bank of simulations and the probability of the delay model was estimated. The process was repeated 10 times. Error bars are the maximum and minimum probability estimated, with the centred dot as the mean. (b) The probability estimate for the probability of the data [47] coming from the delay model as a function of Nthresh. (TIF) [file pcbi.1007930.s009.tif]

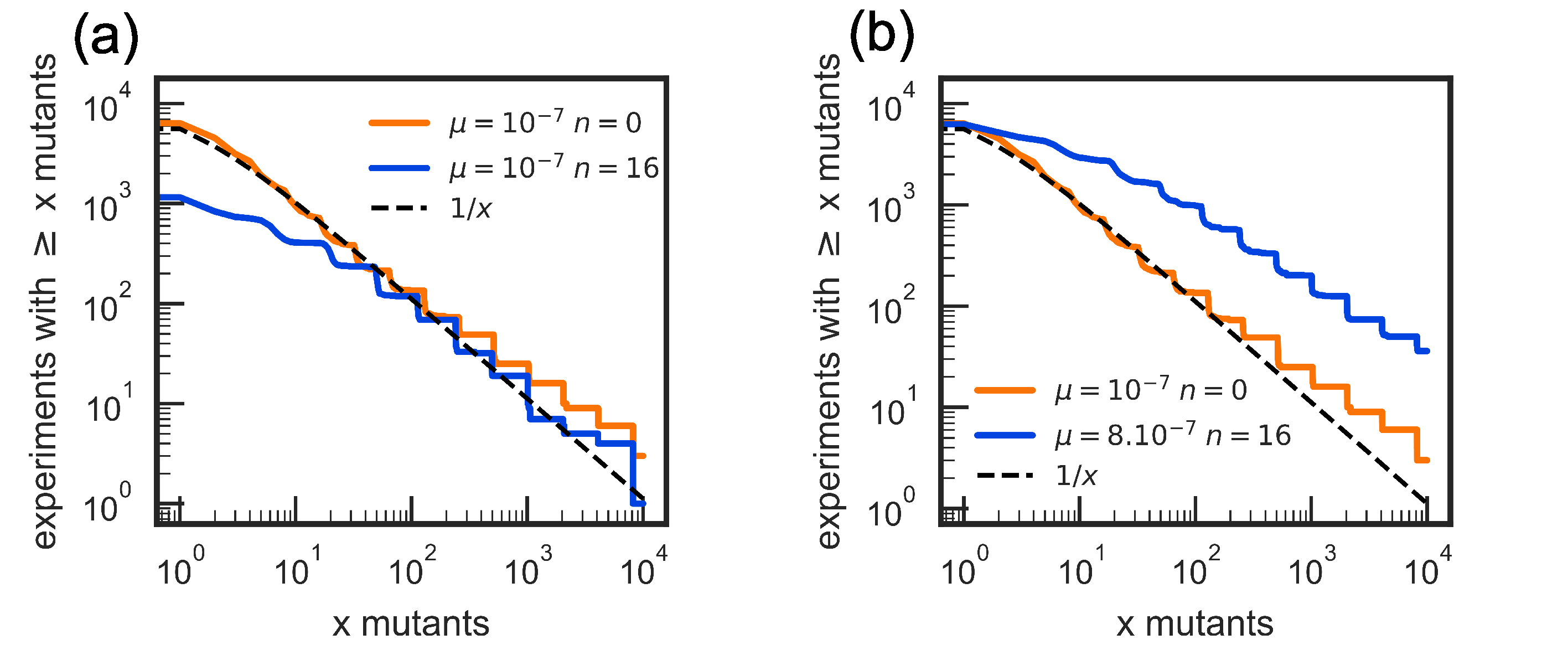

Supplement: S8 Fig — a) Distributions for both models for a fixed μ = 10−7. (b) Distributions for the case when μ in the dilution model has been adjusted to minimize the difference to the no-delay model. (TIF) [file pcbi.1007930.s010.tif]
