# Supplementary material for: Phenotypic delay in the evolution of bacterial antibiotic resistance: Mechanistic models and their implications
Source: PLoS Comput Biol. 2020 May 29;16(5):e1007930. doi: 10.1371/journal.pcbi.1007930 (PMC7307788; doi:10.1371/journal.pcbi.1007930)
Supplement: Attachment — Submitted filename: Phenotypic_delay___cover_letter_and_response.pdf [file pcbi.1007930.s012.pdf]

Editors of *PLoS Computational Biology*

April 25, 2020

Dear Editors,

We thank the Reviewers for their time and comments, and we are glad that they both support the publication of our work. We give detailed responses to each Reviewer's remarks below (Reviewer's comments italicized, our response in plain text).

Reviewer 1 comments

1. *It is not completely clear to me how the dilution mechanism is not a special case of the accumulation mechanism. I have the impression that the two "mechanisms" are effectively only two different parameterizations of the same mechanism, since in both cases, in practice, the cell becomes resistant once the wild-type copies of the proteins are partially/completely removed through successive replication events. My impression is that the difference between the two cases is on how the wild-type copies that are lost upon replication are replaced by resistant copies: in the dilution mechanism it seems that all those lost are replaced in each cell-cycle so that the correct number of total molecules per cell is recovered, while in the accumulation mechanism it seems that only a certain number of resistant copies Mp is produced per cell-cycle. Is there a practical reason to consider these replenishment mechanisms as distinct? I imagine that being production-limited versus total molecule-limited probably depends on the growth rate of the cell, so wouldn't it be more useful to have one parameterization that accommodates both scenarios?*

   We thank the Reviewer for highlighting the similarities between these mechanisms, which we agree should have been mentioned in the text. Our main reason for considering these mechanisms as distinct is to highlight the different effects of different biological mechanisms, e.g. dilution of gyrase molecules vs accumulation of efflux pumps. This leads to the following important difference between the models. In the accumulation mechanism only the number of resistant molecules matters and the number of sensitive ones is irrelevant. The exact opposite is true for the dilution model: the number of sensitive molecules matters whereas the number of resistant molecules is not important.
   In the previous version of the manuscript we wrote that each cell was assumed to have a fixed number of molecules in the dilution model, but this assumption is in fact not

required and was never actually implemented in the simulation code. We have now removed references to a fixed number of molecules, and indicated in the discussion that one could consider the model for molecule production used for the accumulation mechanism as a general molecule production model which could then be specified to the dilution scenario.

2. *Related to the comment about parameterization, it is mentioned how the replication time affects the molecule number n in the dilution mechanism, which is what leads to the dependence of survival probability on doubling time. In order to separate clearly the effects, would it be possible to change the parameterization so that doubling time and molecule number were two independent knobs that could be tuned?*

   We thank the Reviewer for their comment. We have assumed that the number of molecules n in the dilution mechanism depends on the doubling time because this is the case for bacteria (the volume of a bacterial cell increases roughly exponentially with the growth rate, and the concentration of intracellular molecules can also show marked growth-rate dependence). However, as the Reviewer suggests, it is possible to vary n and the doubling time independently in our simulations. In figure S4, we vary the doubling time t_d while keeping n constant, and we observe that the effect on survival probability is minimal (figure S4 panel d). The change in n is thus the primary factor responsible for the change in the survival probability. The residual effect in t_d occurs because faster replication leads to higher ploidy. This in turn reduces the production rate of resistant molecules in the first few generations when only a fraction of the gene copies are resistant.

3. *It is mentioned that both the survival probability at the population level and within a random lineage are measured and reported. However, it would be helpful if the difference between these two quantities in the different range of parameters was discussed in more details, also in relation to lineage experiments, such as mother-machine style, that could be done to investigate the role of phenotypic delay in more details. Since the different mechanisms affect population and lineage survival in different ways, comparing the two could be a way two identify the mechanism at play.*

   We thank the Reviewer for this helpful comment. We agree that the difference between these two quantities is important and was perhaps unclear in the original manuscript. We have now commented in more detail on the relationship between these two quantities and their experimental significance in the Discussion, and added a new panel b to Figure 1.

4. *Finally, for practical reasons, I think it would be helpful to have a table summarizing how most common antibiotics might be affected by these different mechanisms given what we know about their mode of action. This information is already present across the paper, but it would be nice to have it schematically summarized in a table.*

   We have followed this helpful suggestion and summarized this information in the new Table 1.

Reviewer 2 comments

1. *The authors discuss two ways of looking at the problem, per lineage and per population. It is not always entirely clear what exactly the differences are between those two, why they sometimes give different results, and which viewpoint is taken in a given part of the text or figure. For instance, Fig. 1 is concerned with a population where mutations has been introduced, and what is measured is the probability that phenotypic resistance will emerge in that population by some generation post-mutation (it may be helpful to include "post-mutation" to the axis label to reduce confusion that mutation rates are at all involved here). However, the explanation in the text refers to lineages quite frequently, which may be confusing to the readers.*

   We thank the Reviewer for pointing out this potential source of confusion. We have now changed the axis labels from "generation" to "generation post-mutation" in all figures in the main text and supplementary information. We have also included a new panel in figure 1 (panel b in the current version), to clarify the difference between the two ways in which we analyse phenotypic lag: at the single-cell and population level. This difference is also now discussed in more detail in the Discussion section.

2. *In section 2.3 and 2.4, the authors say "this is due to the cancellation of two effects [...]". While the explanation makes intuitive sense, it may improve intuition further to show the expression and the cancellation explicitly to make this point.*

   This cancellation was mathematically shown in Sun et al. (2018). We have now indicated where the derivation can be found in Sun et. al., in Section 2.3.

3. *In some cases of phenotypic delay, the evolutionary dynamics depends on the growth rate. It would be interesting to briefly discuss two more cases: how does individual growth rate variation impact the dynamics, e.g., if the population houses a fraction of slow-growing cells that not only typically have a higher probability of surviving an antibiotic attack by, e.g., betalactams, but that also, per Fig. 3e, have a higher probability of survival due to phenotypic delay. And secondly, how does the dynamics change for the important case of sub-lethal antibiotic concentrations, where growth rates are decreased but non-zero?*

   This is an interesting suggestion, but not an easy one to implement. In our models, we always assume that the antibiotic is present at concentrations which are lethal to the wild type but well below the inhibitory concentration of the resistant mutant. Thus the sensitive population is killed upon exposure, and the emergence of resistant mutants is a stochastic process. The probability of survival of the population is therefore a non-trivial number less than one.

   If, as the Reviewer suggests, we were to model sub-lethal concentrations, the sensitive population (here assumed to be very large) would never die out. The time to resistance would be longer but the effect would be largely due to the overall decrease in growth rate rather than specific details of the mechanism of phenotypic lag discussed in the manuscript. One could also imagine a situation where model parameters such as ploidy, number of target molecules etc., depended on the concentration of the antibiotic. However, we think that this would require modelling a particular experimental scenario, otherwise the discussion would become too speculative. In the current manuscript, we deliberately wanted to focus on idealized models that, while not fully realistic, could provide a basic insight into different mechanisms of phenotypic lag.

Regarding the suggestion about adding slowly-growing cells to the model, the outcome would depend on any possible trade-off between growth rate and resistance. Again, we believe that, since this is highly specific to a particular antibiotic and experimental condition, it is probably better to leave it for future work.

4. *For Fig. 4, it would have loved to see, perhaps as a SI figure, the full distribution on a log-log scale. Does the typical 1/x scaling of the cumulative distribution change, if so, in which regimes and can the scaling be identified? This is roughly shown in Fig. 5b, but a more thorough discussion would be appreciated. Is the staircase-like shape of the simulation line in Fig. 5b a consequence of the non-overlapping generations? This should be explained in the caption.*

   We thank the Reviewer for this suggestion. We have now included the full cumulative distribution on a log-log scale as Figure S8 in the supplementary information. We observe the scaling $\sim x^{-1}$ for both the simulations with and without phenotypic delay, as expected for the Luria-Delbrück distribution. This is now discussed in the results section of the manuscript.

   The staircase-like simulated distributions in figures 5b and S8 are caused by the fixed division times for resistant bacteria and, consequently, their synchronous division. This is now explained in the caption of figure 5.

Sincerely,

Martín Carballo-Pacheco, Michael D. Nicholson, Elin E. Lilja,
Rosalind J. Allen, and Bartlomiej Waclaw
