# Supplementary material for: Phenotypic delay in the evolution of bacterial antibiotic resistance: Mechanistic models and their implications
Source: PLoS Comput Biol. 2020 May 29;16(5):e1007930. doi: 10.1371/journal.pcbi.1007930 (PMC7307788; doi:10.1371/journal.pcbi.1007930)
Supplement: Attachment — Submitted filename: ReviewCarballoPlosCompBio2020.docx [file pcbi.1007930.s011.docx]

The manuscript entitled “Phenotypic delay in the evolution of antibiotic resistance: mechanistic models and their implications” reports a comprehensive theoretical and computational analysis of the role of phenotypic delay in the emergence of antibiotic resistance, with particular focus on three possible mechanisms that can trigger it. The practical consequences on the estimation of mutation rates in fluctuation test experiments are also discussed.

The manuscript is very interesting and its implications important, since fluctuation tests are still the most common method to estimate spontaneous rates of resistance. Its objectives and results are clear and well-explained. I think the manuscript is acceptable as it is. However, I list below some minor comments that I think would improve the manuscript even more if addressed.

1) It is not completely clear to me how the dilution mechanism is not a special case of the accumulation mechanism. I have the impression that the two “mechanisms” are effectively only two different parameterizations of the same mechanism, since in both cases, in practice, the cell becomes resistant once the wild-type copies of the proteins are partially/completely removed through successive replication events. My impression is that the difference between the two cases is on how the wild-type copies that are lost upon replication are replaced by resistant copies: in the dilution mechanism it seems that all those lost are replaced in each cell-cycle so that the correct number of total molecules per cell is recovered, while in the accumulation mechanism it seems that only a certain number of resistant copies Mp is produced per cell-cycle. Is there a practical reason to consider these replenishment mechanisms as distinct? I imagine that being production-limited versus total molecule-limited probably depends on the growth rate of the cell, so wouldn’t it be more useful to have one parameterization that accommodates both scenarios?
2) Related to the comment about parameterization, it is mentioned how the replication time affects the molecule number n in the dilution mechanism, which is what leads to the dependence of survival probability on doubling time. In order to separate clearly the effects, would it be possible to change the parameterization so that doubling time and molecule number were two independent knobs that could be tuned?
3) It is mentioned that both the survival probability at the population level and within a random lineage are measured and reported. However, it would be helpful if the difference between these two quantities in the different range of parameters was discussed in more details, also in relation to lineage experiments, such as mother-machine style, that could be done to investigate the role of phenotypic delay in more details. Since the different mechanisms affect population and lineage survival in different ways, comparing the two could be a way two identify the mechanism at play.
4) Finally, for practical reasons, I think it would be helpful to have a table summarizing how most common antibiotics might be affected by these different mechanisms given what we know about their mode of action. This information is already present across the paper, but it would be nice to have it schematically summarized in a table.
